# Cx43 and the Actin Cytoskeleton: Novel Roles and Implications for Cell-Cell Junction-Based Barrier Function Regulation

**DOI:** 10.3390/biom10121656

**Published:** 2020-12-10

**Authors:** Randy E. Strauss, Robert G. Gourdie

**Affiliations:** 1 Virginia Tech, Translational Biology Medicine and Health (TBMH) Program, Roanoke, VA 24016, USA; 2 Center for Heart and Reparative Medicine Research, Fralin Biomedical Research Institute at Virginia Tech Carilion, Roanoke, VA 24016, USA; 3 Virginia Tech Carilion School of Medicine, Roanoke, VA 24016, USA; 4 Department of Biomedical Engineering and Mechanics, Virginia Polytechnic Institute and State University, Blacksburg, VA 24060, USA

**Keywords:** connexins, Cx43, gap junctions, hemichannels, Zonula occludens 1, barrier function, tight junctions, adherens junctions, actin cytoskeleton, focal adhesions

## Abstract

Barrier function is a vital homeostatic mechanism employed by epithelial and endothelial tissue. Diseases across a wide range of tissue types involve dynamic changes in transcellular junctional complexes and the actin cytoskeleton in the regulation of substance exchange across tissue compartments. In this review, we focus on the contribution of the gap junction protein, Cx43, to the biophysical and biochemical regulation of barrier function. First, we introduce the structure and canonical channel-dependent functions of Cx43. Second, we define barrier function and examine the key molecular structures fundamental to its regulation. Third, we survey the literature on the channel-dependent roles of connexins in barrier function, with an emphasis on the role of Cx43 and the actin cytoskeleton. Lastly, we discuss findings on the channel-independent roles of Cx43 in its associations with the actin cytoskeleton and focal adhesion structures highlighted by PI3K signaling, in the potential modulation of cellular barriers. Mounting evidence of crosstalk between connexins, the cytoskeleton, focal adhesion complexes, and junctional structures has led to a growing appreciation of how barrier-modulating mechanisms may work together to effect solute and cellular flux across tissue boundaries. This new understanding could translate into improved therapeutic outcomes in the treatment of barrier-associated diseases.

## 1. Introduction

Multicellular organisms possess junction-based cellular barriers [1]. Barrier function is a vital homeostatic mechanism characterized by the regulatory exchange of substances between interior and exterior compartments of epithelial tissues, marked by apical and basolateral membrane domains, respectively. Vertebrate epithelial tissue, including its specialized form, the endothelium, is composed of barrier cells, which are defined by the presence of Zonula occludens-containing junctional complexes [2,3]. Pathological stress-induced breakdown in the barrier properties of these junctions is often triggered by a release of inflammatory mediators, which cause dynamic changes in cytoskeletal organization and mechanical force transmission at bicellular and tricellular contact points. These changes result in an unregulated, disruptive exchange and buildup of fluid, ions and other solutes, as well as immune cell infiltration across multiple tissue types and disease processes. Barrier function-associated diseases include Coronary Artery Disease (CAD), Stroke, Acute Respiratory Distress Syndrome (ARDS), Inflammatory Bowel Disease (IBS), and Multiple Sclerosis [4,5,6,7,8,9,10,11]. Barrier-modulating cellular structures include (1) tight junctions (TJ), which provide a gating mechanism that directly controls the exchange of substances across the paracellular space; (2) adherens junctions (AJ), which are critical for the establishment and maintenance of cell-cell adhesion; (3) the actin cytoskeleton, which controls the overall integrity of cell-cell contacts via mechanical push/pull forces; and (4) gap junctions (GJ), which allow for exchange of signaling molecules and ions between cells through connexin-based transcellular channels, in addition to close points of intercellular adhesion. While traditionally considered to be independent structures, evidence indicates a high degree of crosstalk amongst these different cellular complexes, including via a shared, direct interaction with the tight junction scaffolding molecule, Zonula occludens 1 [12,13,14,15].

There is growing appreciation that the gap junction protein subunit, Cx43, has both channel-dependent and -independent functions. This evidence indicates that Cx43 influences cell-cell contact arrangements and cytoskeletal dynamics, junction assembly, cell polarity, and transcriptional regulation [16,17,18,19,20]. Findings have emerged over the last 20 years or more that connexins, especially the most studied isoform, Cx43, are involved in regulating barrier function and permeability. This review will focus on the crosstalk between Cx43, the actin cytoskeleton, and the various transinteracting junctional complexes in the regulation of barrier function. Throughout this text, the barrier-relevant effects of Cx43 mimetic peptides will be highlighted as evidence of such crosstalk. Recent developments on the role of connexins in barrier function enrich our understanding of the fundamental biology of this important class of molecules. Moreover, this new information provides insight on disease mechanisms and potential novel therapeutic approaches to the treatment of barrier-associated pathologies. 

## 2. Connexin Structure and Function

### 2.1. Cx43

**Figure 1:**
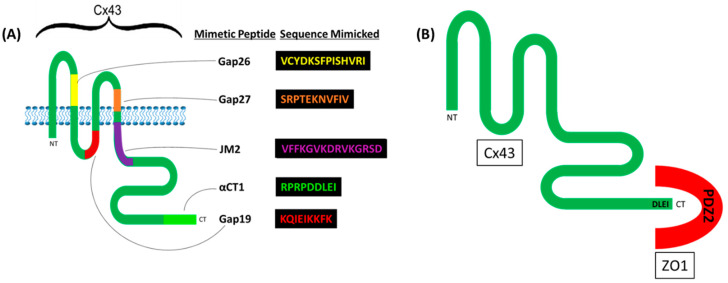
Cx43 structure and the amino acid sequences contained within Cx43 mimetic peptides. (**A**) Cx43 contains four conserved α-helical transmembrane domains, two extracellular loops, a cytoplasmic loop, and cytoplasmic amino (N)- and carboxyl (C)-terminal domains. Cx43 mimetic peptides mimic Cx43 function by containing sequences identical to those found within these domains. (**B**) Cx43′s PDZ2-binding ligand (DLEI), contained at the terminal-most portion of its CT, inserts into the PDZ2 domain of ZO1.

To date, 21 different connexin genes have been identified in the human genome, though only Cx37, Cx40, Cx45 and Cx43 have been found in the vascular endothelium specifically [21]. The most ubiquitously expressed isoform is Cx43 [22]. Cx43 is a 382 amino acid (aa) long polypeptide comprised of four conserved α-helical transmembrane domains, two extracellular loops, a cytoplasmic loop, and cytoplasmic amino (N)- and carboxyl-terminal (CT) domains. The N-terminus (NT) is relatively short (13 amino acids), while the CT is relatively long (w/150 amino acids). Multiple regions along its length have been utilized as peptide sequences for a variety of Cx mimetic peptides such as αCT1, Gap19, Gap26, and Gap 27 (Figure 1A). The C-terminal (CT) of Cx43 (as well as other connexins) is highly disordered, flexible, and modifiable in its interactions with tight junction adaptor Zonula occludens 1 (ZO1) [23,24,25].

Cx43 exhibits a high-affinity interaction with the PDZ 2 (post-synaptic density protein (**P**SD95), Drosophila disc large tumor suppressor (**D**lgA) and Zonula occludens 1 (**Z**O1)) domain of ZO1 [26] (Figure 1B). In 2008, the crystal structure of the interaction between Cx43′s CT and ZO1 was solved by Chen and colleagues [27]. In this structure, domain-swapped PDZ2 domains of ZO1 contain a groove, a hydrophobic pocket into which hydrophobic side chains of the CT of partner proteins such as Cx43 insert, allowing for a high-affinity interaction. Via similar PDZ2 binding motifs(DLXI) in their carboxyl termini, five or more different connexin isoforms have been shown to interact with the ZO1 PDZ2 domain in this manner, including Cx50, Cx46, Cx43, Cx31.9, and Cx30. Cx35/36 and Cx45 interact with the PDZ1 domain of ZO1 [28]. The terminal isoleucine within Cx43 CT’s class 2 PDZ binding motif is critical for this interaction [29]. Such binding characteristics between the CT-most region of Cx43 and the PDZ2 domain of ZO1 has been corroborated by Jiang and colleagues with the use of the Cx43 mimetic peptide, αCT1, containing the CT-most 9 aas of Cx43 (RPRPDDLEI) (Figure 1).

Connexins combine to make larger structures with channel properties traditionally associated with connexin function. Six connexins make up a connexon (also known as hemichannel). Disulfide bonds between the extracellular loops of connexin transmembrane domains enable interactions amongst connexin subunits within the connexon via highly conserved cysteine residues. This bonding allows for the formation of a channel pore when connexin subunits are oligomerized in canonical hexameric fashion [25].

Connexon-containing vesicles transport from the ER/Golgi network along microtubules to the membrane in oligomerized form [30,31]. Homo/heterophilic interactions between hemichannels (HCs) across the paracellular space constitutes a GJ channel (GJC). Thus, this structure is characterized as a cell-cell conduit composed of closely adjoined, hexameric transmembrane spanning protein complexes interacting across the paracellular gap [31,32]. A cluster of these intercellular channels is called a gap junction (GJ), and when aggregated in this manner, these structures span a 2–4 nm intercellular space or “gap” [33]. This is a uniquely small distance between two membranes, where the cell-cell apposition is found to be tighter only at the *tight* junctions [34,35], the structures which we will discuss shortly.

Multiple phosphorylation sites, particularly at serine residues (e.g., S373 and S368) within the connexin CT, have been shown to regulate GJ formation by modulating the binding to, and release from, ZO1 [36,37]. Upon arrival at the membrane, connexons are thought to dock to ZO1 at the perimeter of the GJ plaque, a location termed the “perinexus” by Gourdie and colleagues [38,39]. Phosphorylation on S373 has been shown to facilitate GJ accretion by altering Cx43′s conformation, resulting in uncoupling between the CT and ZO1. As a result of this process, HCs travel medially into the GJ plaque to dock with connexon neighbors on the membrane of the opposing cell. In fact, this effect mimics the well-characterized property of αCT1, as it was designed to directly inhibit the Cx43 CT/ZO1 interaction [38,40,41]. 

### 2.2. Canonical Channel-Dependent Functions of Cx43

The canonical channel-dependent functions of GJs fall into two main categories: ionic/electrical conduction via exchange of Na^+^, K^+^, Ca^2+^ ions, and molecular coupling of metabolites (ATP/ADP, glucose, glutamate, glutathione) and secondary messengers (IP3, cAMP) between cells [33,42,43,44]. In either case, the primary function of GJs is to provide a conduit for the direct transfer of material between cells. Such functions allow for direct and indirect functions in electrical and molecular signaling across cell monolayers and tissues (e.g., for the synchronous beating of the heart). Opening and closing of GJ channels is regulated by factors that include post-translational modification (e.g., phosphorylation), chemical (e.g., Ca^2+^ and pH) gating and transjunctional voltage changes [45,46]. GJ channels have notable roles in the propagation of injury signals via exchange of ATP, cAMP, glucose, Na^+^ and other metabolites and ions across an extensive range of disease processes. Cx43-based gap junction intercellular communication (GJIC) has been extensively studied in processes such as wound healing [47] and cardiac conduction [48,49,50]. Important to note, however, is that the dominant role of GJIC in function of the heart has been met with rising skepticism by Gourdie and colleagues. A review by Gourdie [51] reported on what was termed a “mixed mode” of cardiac conduction, underlying the beating of a healthy heart thought to involve both electrotonic GJIC and an ephaptic mode of communication (originally identified in nervous tissue). The latter mode is highlighted by transcellular voltage regulation by the transinteracting beta-1 (β1) subunit of the voltage-gated Na+ channel. See other reviews on this topic [50,52].

HCs have some overlapping functions with GJs, in that they exchange many of the same types of substances (e.g., ATP and Ca^2+^), and facilitate the propagation of injury signals, although the substance exchange is not spatially limited to the interior space of the cells [53,54]. Instead, HCs provide a conduit for information exchange between the interior of the cell and the extracellular space. Several factors, including changes in intra- and extracellular Ca^2+^ levels, mechanical forces, metabolic inhibition, redox potential, interaction with protein partners (e.g., ZO1), as well as post-translational modifications regulate the individual and en masse opening of HCs [53,55,56]. In a mutually exclusive manner with ZO1-binding, HC opening appears to require intramolecular interaction between the CT of the connexin subunits and the cytoplasmic loop domain [57]. One of the most established mechanisms regulating this event is changes in intra/extracellular Ca^2+^ levels—either a decrease in extracellular or increase in intracellular Ca^2+^ result in large-scale HC opening [58]. Unregulated opening of HC has been implicated in a variety of disease processes such as heart disease, skin diseases and deafness [53].

### 2.3. GJs and Cell–Cell Adhesion

Gap junctional structures have cell-cell adhesive characteristics [59,60]. The homo/heterophilic interactions between HCs across the paracellular gap must provide a source of cell-cell adhesion if by no other measure than the summative strength of the transcellular interaction between extracellular loops of apposed connexons. A nice example of this was demonstrated by a study performed by Elias and colleagues [61]. They showed that connexin-based GJs are highly expressed at radial glial fiber cell-cell contacts, particularly at radial fiber endpoints and that adhesive properties of GJs are necessary for radial migration. In this study, shRNA knockdown of either Cx36 or Cx43 via in utero intravenous injection of Sprague-Dawley rats, produced defects in neuronal migration. To determine the mechanism behind the neural migration defects, they introduced mutations in the connexin targeting shRNAs, which rescued normal migration, then incorporated manipulations involving inhibition of electrical or chemical GJ coupling. Neither constitutive inhibition of channel activity via a mutation of conserved tyrosine on third transmembrane domain of Cx26 or Cx43 (Cx26CMT135A or Cx43CMT154A), nor inhibition of purinergic-based Ca^2+^ wave propagation by knockdown of P2Y1 receptor, showed defects in migration. However, when one of the mutations of the conserved extracellular cysteines (Cx43CMC61S) to produce adhesion-incompetent GJs failed to rescue the migration defect, the authors concluded that it was the adhesive properties of GJs underlying the mechanism of radial migration defects.

GJIC is evident across a wide range of disease processes, which characteristically involve the breakdown of bona fide adhesive structures such as AJs and TJs [2]. Thus, it is plausible that GJs may be one of the few, if not the only, cell-cell adhesive structures that on occasion remain during breakdown of cell-cell contacts.

### 2.4. Connexins and Barrier Function

Evidence has accumulated over the last two decades implicating non-canonical roles for connexins in diseases associated with impairments in barrier function. This is a pathological correlate of a variety of diseases such as CAD, Stroke, ARDS, IBS, as well as Multiple Sclerosis [6,7,62]. Indeed, via an interaction with ZO1, connexins exhibit crosstalk with multiple barrier-modulating structures, including tight junctions and the actin cytoskeleton [13]. However, before discussing the emerging role of connexins in barrier regulation in detail, we will first outline the key structures involved in barrier function regulation.

## 3. Barrier-Modulating Structures

### 3.1. Tight Junction Structure and Function

The TJ forms a transinteraction across the paracellular space to directly control substance exchange. TJ proteins claudins and occluding (as well as connexins and innexins) are members of a Tetraspan (4 TMS) Junctional Complex (4JC) Superfamily (4TMS 4JC) [63]. This family of proteins possesses four transmembrane spanning α-helical (TMS) topologies and exhibit well-conserved extracytoplasmic cysteines that can form extracellular disulfide bridges involved in homomeric transcellular interactions. In freeze-fracture electron microscope en-face images, these junctions appear as long intertwining fibrils of intramembrane particles located apically along the exoplasmic face of each cell [64]. TJs confer upon tissues a physical and charge-, ion- and size-selective barriers via a “gate” function. TJs moreover assist in maintaining apical-basolateral polarity by differentiating between fluid compartments across the tissue apical-basolateral axis via their “fence” functions [65,66]. Although built from >40 different proteins, there are two general classes of TJ protein: those that impose barriers to free diffusion through the paracellular space (e.g., occludin and some claudins); and those that exhibit channel-like properties, known as the pore-forming junctions. These latter classes are comprised exclusively of claudin isoforms and correspond to a “pore” pathway for paracellullar flux, which is size and charge selective. This “leak” pathway is relatively non-discriminatory—theory suggests that it is undergirded by a large scale breaking and reannealing of TJ strands.

All of the barrier-modulating structures discussed in this review exhibit interactions with the TJ-scaffolding protein ZO1. ZO1 belongs to the MAGUK superfamily (Membrane-Associated Guanylate Kinase) and is present only in multicellular organisms. This molecule in its discovery, located at the space where two opposing membranes come closest together, was viewed as an occlusion of the extracellular space, so was named Zonula occludens, which means closing belt in Latin. ZO1 is a peripheral membrane protein that is cytoplasmically located on the luminal (apical) side of the epithelial monolayer, forming a continuous belt that wraps around a large portion of the cell’s perimeter [3,67]. Zonula occludens belongs to a protein family comprised of three isoforms, ZO1, ZO2, and ZO3, encoded by *TJP1, TJP2, TJP3* genes, respectively, named by the order of their discovery [64]. 

**Figure 2:**
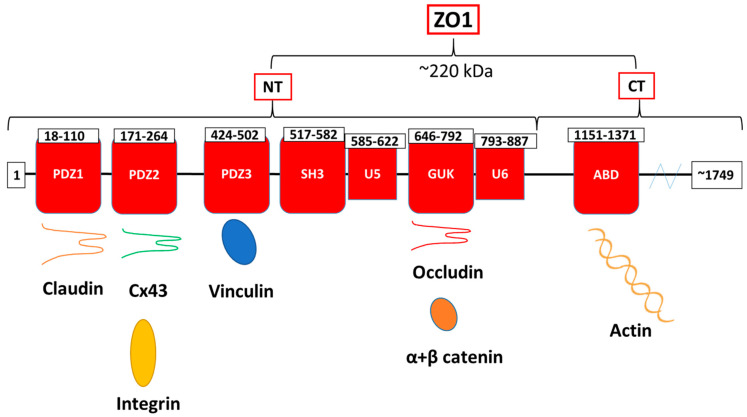
TJ adaptor ZO1 structure and its interacting partners in barrier function regulation. ZO1 consists of three PSD95, DlgA, ZO1 homology (PDZ) domains (PDZ 1, PDZ 2, and PDZ 3), a SRC homology 3 (SH3) domain, a yeast Guanylate Kinase homology (GUK) domain, and Unique-5 and -6 motifs (U5, U6) within its NT half, and an actin-binding domain (ABD) within its CT portion.

ZO1 is by far the most studied and well characterized of the three isoforms. This 220 kDa protein includes three PSD95, DlgA, ZO1 homology (PDZ) domains (PDZ 1, PDZ 2, and PDZ 3), a SRC homology 3 (SH3) domain, a yeast Guanylate Kinase homology (GUK) domain, and Unique-5 and -6 motifs within its NT half, and an actin-binding domain (ABD) within a CT-located domain [68,69] (Figure 2). ZO1 possesses a distinct capability to interact with numerous other binding partners, a feature conferred upon it by its diverse set of protein-protein interaction domains. ZO1 thereby crosslinks with a host of junctional complexes, other members of the ZO family (ZO2 and ZO3 isoforms), focal adhesion complexes, and the cytoskeleton of the cell. These junctional complexes mostly interact with ZO1 within its NT half-comprising in turn its PDZ3, SH3, U5 and GUK domains. For example, many members of the tight junction protein family of claudins bind to the first PDZ (PDZ1) domain [70], whereas connexins mostly interact at the second PDZ domain (PDZ2) [14,28] The focal adhesion protein vinculin binds to the third PDZ domain (PDZ3) [71] and occludin [72] and AJ molecules [69,73,74] interact with the GUK domain (Figure 2). Interestingly, ZO1 is force sensitive and is considered to be a “tension transducer”, exhibiting tension-dependent intramolecular interactions between its NT and CT portions [75]. Under actomyosin contraction, tension is created between the membrane-anchored tetraspanins bound to the NT portion and the actomyosin apparatus and actin-binding proteins (e.g., vinculin and α-catenin) bound to the CT portion. As a result, ZO1 can take on an open/closed conformation, regulating access to its central SH3-U5-GUK-U6 region to control protein binding (e.g., with occludin) and targeting signals (e.g., localization to the TJ complex) [76]. 

### 3.2. Occludin

The first integral TJ protein to be identified, in 1993 [77], once regarded as the “holy grail” of TJs, was occludin, assigned the name “occludin” from the Latin word “occlude” [78]. As determined by electron microscopy, these occludin-based fibrils tightly interact between two cells at the junction points along their length, forming what is known as “kissing points”, to the near absolute exclusion of the extracellular space [79]. Occludin has been found to play a significant role in barrier function across multiple tissue and cell types in epithelium and vascular endothelium of brain, heart, liver, kidney, intestine and many other organs. However, since its identification, multiple studies have shown that it is neither sufficient for TJ strand formation nor vital to life [80,81].

In the mid-20th century, barrier-forming structures occurring at tricellular contact points were discovered. Electron microscopy showed that as occludin-based TJs approach tricellular contact points, the TJ extends basolaterally at the exoplasmic face of each cell, as a vertically oriented triple pair strand structure, forming a “central tube” of approximately 1–10 nM in diameter [82,83,84]. This description characterizes the tricellular junction complex. Phylogenetic analysis places the tricellular junction protein, tricellulin, as a tetraspanin in the same family as occludin [85,86]. Although the extent to which tricellular versus bicellular junctions are involved in barrier function has yet to be established under tricellulin-deficient conditions, evidence shows that overexpression of tricellulin, reduced paracellular permeability to macromolecules [87]. The evidence indicates that some macromolecule leak occurs primarily at the tricellular junction, an alternative to the better known hypothesis explaining paracellular permeability (dynamic breaking and reannealing at bicellular contacts of the TJ).

With a molecular weight of approximately 65 kDa, studies have shown that occludin, particularly its NT-located extracellular loop, is involved in regulation of neutrophil transmigration, extrusion of apoptotic cells from cell monolayers via Rho activation, and adhesion at cell-cell contacts [1,88]. The function of occludin, among others related to barrier maintenance, requires its ability to bind to the GUK domain of ZO1 at the cytoplasmic plaque [89].

### 3.3. Claudin

Claudins bind to the first PDZ (PDZ1) domain of ZO1. With their discovery and identification in a purified junctional fraction from chicken liver, came the experimental determination that the claudins were critically responsible for paracellular barrier function as they alone were sufficient to form TJ strands [90]. Claudin-null mice die hours (10 h) after birth resulting from hyperpermeability in the blood brain barrier [91]. Claudins are 20–24 kDa proteins comprised of a family of 27 four-transmembrane domain proteins in mammals, which are mainly responsible for the maintenance of paracellular permeability in epithelia.

Claudins are much more diverse than the occludins, and create paracellular barriers across a relatively greater spectrum of characteristics. In contrast to occludins, claudins additionally possess channel properties highlighted by ion and size-selectivity, thus constituting the “pore” pathway. A subset of claudins (e.g., 2, 10b, 15, 16, and 21) are anion selective (e.g., allowing negatively charges ions to pass) and others are selectively permeant to cations (e.g., 10a and 17). Interestingly, studies have shown that Claudin-2 forms a water channel, mediating the paracellar transport of water in leaky epithelia [92,93]. In the brain, claudin-5, a strictly barrier-forming as opposed to a channel-forming claudin, plays a critical role in maintaining the blood-brain barrier and is the most well-studied and essential claudin for barrier function in endothelial cells [94].

### 3.4. Adherens Junctions

Cadherin molecules of both epithelial and endothelial tissue mediate homophilic, Ca^2+^-dependent trans (and cis) interactions via their extracellular immunoglobulin domains [95,96]. AJs are thought critical for the establishment and maintenance of cell-cell adhesion and are important for barrier function regulation, particularly in endothelia [97]. Experiments suggest that these structures are necessary for TJ formation, but are not required to maintain TJ integrity. During cell-cell contact maturation in epithelial tissue, TJ formation is preceded by AJ stabilization [98,99]. AJs, and possibly other junction complexes, exhibit a catch-bond nature by increasing their strength in response to increased tensile force. However, mathematical modeling studies showed that junctional breakage and opening of free spaces (intercellular gaps) will form when forces are increased beyond the maximal lifetime of a single molecular bond, resulting in loss of barrier function [100,101].Through their cytoplasmic domain, cadherins (VE-cadherin in endothelial, or E-cadherin in epithelia) directly bind to catenins (p120-catenin, β-catenin and α-catenins) and these associations stabilize ZO1 binding and appear to be critical for the maintenance of AJ integrity [102]. 

### 3.5. Actin

Scaffolding proteins such as ZO1 and Vinculin have been shown to mediate the interaction between α-catenin and the actin cytoskeleton for the transmission of actomyosin push/pull forces at TJ-based barriers [76,103,104,105]. Actin contractile structures may contract radially or parallel to the plasma membrane, as cells tethered to neighboring cells by cell-cell junctions can dynamically form and break in a force-dependent manner [5]. The actin cytoskeleton takes on two general forms—a cortical formation, characterized by a peripherally located circumferential band and a parallel filamentous (F-actin) stress fiber (SF) arrangement, which spans the cytoplasm. Dynamic interchange of these two significant structures, accompanied by continuous and discrete polymerization/depolymerization processes, myosin-driven contractile forces, and changes in cellular distribution, contribute to the role of actin in intercellular gap formation and barrier function control [106,107].

**Figure 3:**
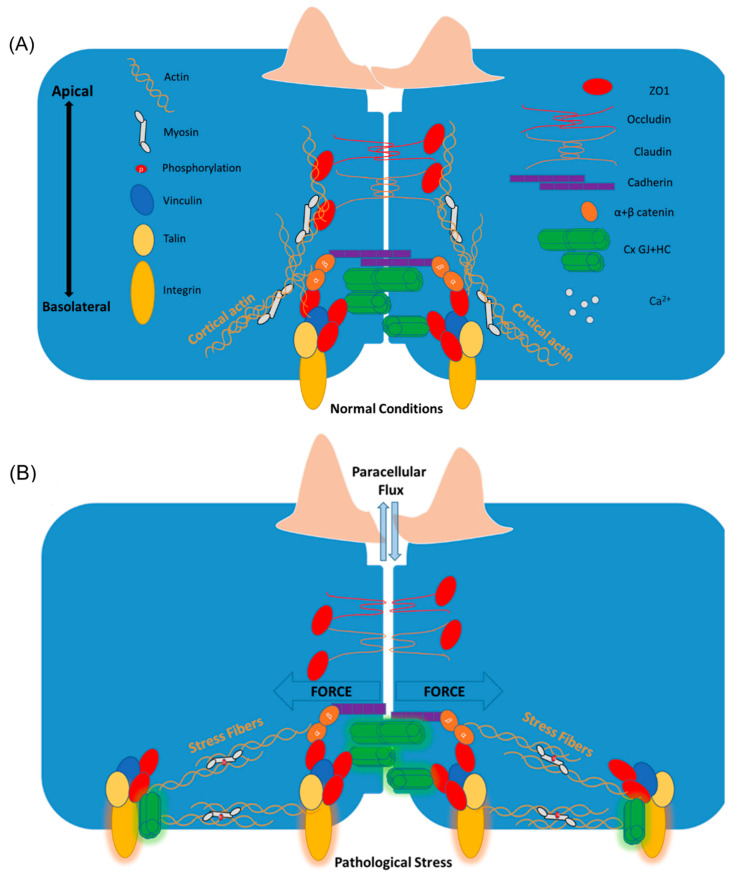

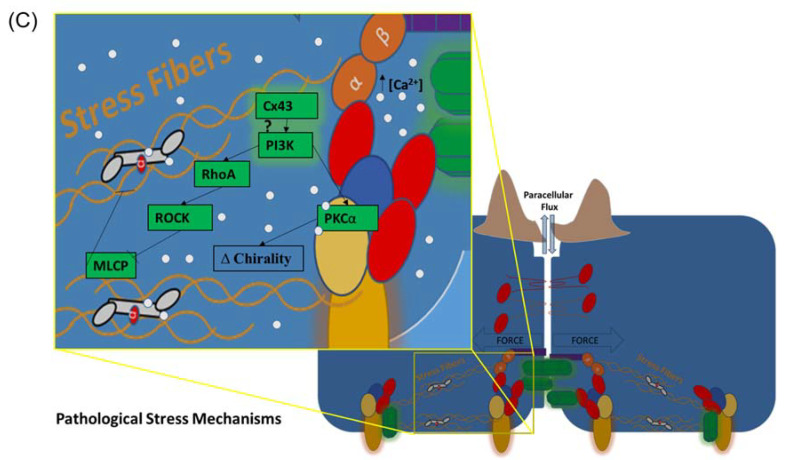
A proposed integrative model for channel-dependent and -independent roles of Cx43 in actin-mediated barrier function pathology. (**A**) A barrier-stabilizing molecular network under normal conditions, including Cx43, focal adhesions, branched cortical actin structures, and transcellular interacting junctional complexes. (**B**) A dynamic shift to actomyosin SF formation, accompanied by upregulation and activation of focal adhesion structures under pathological stress conditions. These processes result in force transmission to AJ-based cell-cell contacts and a breakdown in TJ-regulated barrier function marked by unregulated flux of solutes and ions across the paracellular space. (**C**) Channel-dependent and -independent roles of Cx43 and the Cx43/ZO1 interaction in the stabilization and alignment of actomyosin pulling forces exerted onto membrane-linked focal adhesion and AJ structures. Under pathological stress conditions, Cx43-based GJs and HCs support Ca^2+^ flux, while Cx43 signals via PI3K (by an unknown mechanism) to activate Ca^2+^-dependent SF contraction and PKCα-mediated changes, resulting in a cell chirality-associated breakdown of barrier function.

Under normal conditions, actin takes the form of a cortical actin rim, running parallel to cell-cell borders, encircling the inner cytoplasmic face of the membrane. Cortical actin exerts centrifugal (outwardly) directed tension, which opposes centripetally (inwardly) directed inward contractile forces of the SF phenotype, which will be discussed shortly [108]. The cortical actin network comprises short, branched, intersecting fibers at the cell periphery, where the TJ protein ZO1 is concentrated. This actin “rim”, bound to ZO1, stabilizes cell-cell and cell-matrix interactions by tethering these structures to intracellular components, thereby contributing to the stability of the tissue barrier [109,110] (Figure 3A). Belardi and colleagues [68] recently reported that a 28 amino acid actin-binding site (ABS) within the CT half of ZO1 was required for maintaining transepithelial resistance (TEER) and permeability using both impedance-based and macromolecular permeability based assays for barrier function measurement. Sphingosine-1 phosphate (S1P) and cAMP analogues stimulate peripheral actin formation, involving cortactin-regulated, PI3K/AKT/Rac-mediated generation of local membrane projections, termed lamellipodia, which reduce the formation of intercellular gaps and strengthen barrier function during barrier function maintenance and repair [111,112,113]. 

Cell stressors, such as thrombin and other inflammatory mediators or conditions (e.g., histamine, lipopolysaccharide, endotoxin, Tissue Necrosis Factor (TNF), and shear stress), can shift the balance of actin away from a cortical phenotype to the formation of RhoA/ROCK/pMLC-regulated SFs [114]. These filaments span the cell and in a sarcomeric-like manner, prompt cell contraction and the breaking down of cell-cell contacts—a change that promotes leukocyte infiltration and fluid leakage into tissues [115]. Contraction of actomyosin is associated with a breakdown of AJs and TJs, and a redistribution of their respective proteins away from sites of cell-cell contact [116,117,118]. One study showed that the actin depolymerizering agent, Latrunculin A, blocks thrombin-induced SF formation, endothelial cell retraction and barrier disruption [119]. Two major actin SF classes (ventral and dorsal) run orthogonal to the cell boundary and are nucleated at, and anchored by vinculin-containing focal adhesions [118,120].

### 3.6. Vinculin-Based Focal Adhesions

Focal adhesions anchor cells to the extracellular matrix, and transmit mechanical and regulatory signals to and from the cell. Vinculin, via its NT-located globular head, binds ZO1, at the PDZ3 domain of ZO1. The CT of vinculin contains two critical F-actin-binding residues V1001 and I 997 [121]. Furthermore, vinculin interacts with the focal adhesion protein, talin, which anchors vinculin to the integrin-based cell-matrix contact [122]. As discussed later, vinculin-containing focal adhesion complexes are maintained by the presence of Cx43.

Vinculin-based focal adhesions are the sites of Rho-mediated SF nucleation, anchorage, alignment, elongation and branching [123,124]. One study showed that regions of high focal adhesion tension had highly aligned linear actin filaments, whereas regions of low tension had less well-aligned F-actin [125]. Another study showed that focal adhesion tension was lower in cells on soft substrates, in keeping with the phenomenon of cellular stiffness sensing, in which soft surfaces trigger a reduction in contractility [126]. Experiments have shown that vinculin is critically involved in contractile-dependent mechanisms of endothelial permeability by integrating focal adhesions with SFs [127,128]. When anchored to a stiff extracellular matrix (ECM) via vinculin attachment, F-actin SFs transmit radial mechanical tugging forces that pull centripetally on ZO1, catenins, and vinculin complexes at bicellular and tricellular contact points. This process results in a disruption of transcellular interactions and opening of leaky intercellular gaps within barrier cell layers [4,5,127]. As we will discuss later, potential channel-dependent and -independent roles of Cx43 in barrier function regulation may involve a ZO1-mediated link to focal adhesion complexes.

## 4. Channel-Dependent Roles of Connexins in Barrier Function

### 4.1. The Role of GJIC in Barrier Function Regulation

A number of studies indicate the deleteriousness of connexin-based GJIC on barrier function, particularly in the endothelium [10]. Both GJs and HCs have been shown to mediate pathology triggered (e.g., histamine, hypoxia, thrombin, and bradykinin) rises in intracellular [129] and Ca^2+^ wave propagation associated with endothelial barrier breakdown. In line with this, GJ and HC gating have been correlated to actomyosin contractility and SF formation, processes associated with Ca^2+^-dependent mechanisms (e.g., myosin II-associated MLC phosphorylation) [130,131,132,133,134]. In a model of pulmonary edema, O’Donnell and colleagues [135] showed that treatment with the inflammatory agents, thrombin and LPS, increased mRNA and protein levels of Cx43, and increased GJIC. In this study, pretreatment with GJ inhibitor, carbenoxolone, as well as Cx43 siRNA knockdown, attenuated thrombin- and LPS- induced barrier function disruption, and thrombin-induced phosphorylation of myosin light chain (MLC), in endothelial monolayers. Another study by Zhang and colleagues [136] reported that vascular permeability increased in the lungs, kidneys, and mesentery in septic rats and in LPS-stimulated monolayers of pulmonary vein endothelial cells. These disruptions positively correlated with expression of Cx43 and Rock1. Consistent with the role of GJIC in modulating Ca^2+^-dependent SF formation and contraction, both a Rock inhibitor and carbenoxolone, protected the tissues from vascular leakage. Overexpression of Cx43 increased the phosphorylation status of MLC and the expression of Rock 1, which was reversed with Cx43 siRNA knockdown. Addition of endothelin-1, shown to enhance Cx40- and Cx43-based GJIC [137], caused an increase in F-actin SF formation in astrocytes, which was shown to be largely dependent on the presence of Ca^2+^ in the media [138]. Lastly, in a model of familial cerebral cavernous malformations type III (fCCM3), CCMD KO mice, Cx43 expression increased within lesions, formed large GJ plaques, and increased GJIC in CCM3KD cells [139]. The GJIC inhibitor Gap27 rescued CCM3KD hyperpermeability, causing a disbanding of GJs. 

On the other hand, multiple studies have indicated barrier-protective roles of GJs. One study showed that the addition of GJ blockers 18β-glycyrrhetinic acid (18β-GA) and oleamide (OA) reduced the barrier function of blood-brain barrier (BBB) endothelial cells, as indicated by a drop in TEER, yet curiously did not affect expression of Cx40, Cx43, occludin, Cl-5, ZO1 or other tight junction-associated molecules [140]. Interesting to note is that this study reported on GJIC mechanisms revealed under normal conditions, rather than pathological conditions. Another study showed that mice lacking either Cx37 or Cx40, the predominant connexins present in vascular endothelium, are viable and exhibit phenotypes that are largely non-blood vessel related. However, animals lacking both Cx37 and Cx40 display severe vascular abnormalities, with localized hemorrhages in skin, testis, gastrointestinal tissues, and lungs, and die perinatally [141]. While this study suggests barrier-modulating properties of connexins, whether or not GJIC was mechanistically involved in the generation of vascular malformation was not elucidated.

While these and other studies indicate a barrier-protective role of GJs [142,143,144], many of these same reports have not conclusively determined that changes in barrier function occurred as a direct result of changes in GJ channel properties. For instance, the role of connexon-connexon interactions as potential adhesive structures participating in cell-cell contact integrity is often not considered. Moreover, no studies to date have measured the barrier-altering effects of channel loss-of-function as rendered by GJIC-specific genetic mutations. Lastly, many of the studies reporting on the protective roles of connexins in barrier function, were conducted under normal conditions, while leaving open the question of what occurs under barrier-disruptive conditions. Future experiments might parse the relative contributions of connexin-based interactions as adhesive structures versus intercellular channels in the modulation of barrier function under both normal and pathological conditions.

### 4.2. The Role of HCs in Barrier Function Regulation

In line with the deleterious effects of GJs, the unregulated opening of HCs has generally been associated with barrier disruption [131,132,145]. A wide range of disease processes involve intracellular rise in Ca^2+^ triggering en masse opening of HCs [58]. Connexin HCs provide a pathway for Ca^2+^ entry into the cell [146], which is necessary for sustained intercellular Ca^2+^ oscillations [147]. De Bock and colleagues have shown that HC-mediated rises in intracellular Ca^2+^ can trigger actomyosin contraction, and in a paracrine-like manner, ATP release can cause similar effects via purinergic receptor signaling in nearby cells [132,148,149,150]. One set of experiments showed that inhibition of HCs and GJs using high concentrations (200 µM) of Gap27 prevented endothelial hyperpermeability induced by low extracellular Ca^2+^ [130]. This protection was thought to override the destabilizing effects on the Ca^2+^-dependent AJs. Another study by De Bock and colleagues [151] showed that Bradykinin (BK)-induced BBB hyperpermeability and Ca^2+^ oscillations were blocked by carbenoxolone, Gap27, and Cx37/43 knockdown. The organization of the cytoskeletal intermediate filament protein, vimentin, was protected by addition of HC blockers. While ATP treatment alone induced Ca^2+^ oscillations in this study, it did not induce HC opening nor a disruption of endothelial barrier function. Moreover, ATP-release induced by BK was not associated with the timing nor phase of Ca^2+^ spikes in nearby cells. Thus, the authors determined that it was the targeted inhibition of ATP-independent Ca^2+^ oscillations by HC blockers, which facilitated barrier function protection.

Inhibiting Cx43 HCs with the Cx43 mimetic peptide, TAT-Gap19, protected mice against TNF-induced mortality, hypothermia and vascular leakage, while a 15 min preincubation with TAT-CT9 provoked opposite effects [152]. It should be noted that the TAT-CT9 peptide is identical to αCT1, but the 9 aa Cx43 CT sequence (RPRPDDLEI) is instead attached to a TAT cell penetration sequence as an alternative to the αCT1-attached antennapedia. While in this study, TAT-CT9 facilitated HC opening, one could infer that αCT1 affects HC gating similarly, an effect in apparent contradiction to the barrier-protective role exhibited by αCT1 as will be discussed shortly. However, experiments performed by Gourdie and colleagues indicate that αCT1 induces a downregulation in overall HC activity while facilitating GJ accretion [38]. Furthermore, αCT1′s induction of S368 phosphorylation was found 6 hrs post-treatment of injured myocardial tissue, a phosphorylation event linked to HC closure [29,153,154,155]. Thus, consideration of Cx43-modulating peptides require careful attention to the timing and context-dependency of their effects.

Taken together, a common mechanism linking Cx43-based HC and GJ function to barrier properties is the channel-dependent modulation of intracellular Ca^2+^ levels, accompanied by changes in the cytoskeleton. While evidence is consistent across studies on a barrier-disruptive role of pathology-triggered HC opening, the role of GJIC in barrier function is less clear. Important to note is that many of the chemicals used to block GJ function have shown ability to block HCs as well [156]. Thus, future studies should assess cytoskeletal changes associated with barrier-modulation, while specifically investigating and discriminating between the channel-dependent functions of HCs and GJs.

## 5. Channel-Independent Roles of Cx43 in Barrier Function

### 5.1. Key Studies 

Even less understood is the channel-independent role of Cx43 in the regulation of barrier function. Only a few studies to date have revealed direct evidence of the contribution of connexins to barrier modulation, independent of their channel properties. One of the first studies on this topic showed that mice lacking the Cx43 CT die early due to epithelial barrier dysfunction, while exhibiting normal GJIC [157]. This raised the possibility that the CT of Cx43, including via its high-affinity interaction with ZO1, might be involved in barrier function homeostasis. Using classical and automated in vitro methods of measuring resistance/impedance across cell monolayers, Obert and colleagues [158] showed that αCT1, prevented VEGF-mediated breakdown of the TJ-based barrier in a channel-independent manner. In this study, Cx43-expressing ARPE-19 cells were pretreated with 30–100 µM αCT1. The VEGF-induced drop in barrier function, as measured by TEER, was significantly attenuated in αCT1-treated monolayers. The GJ blocker, 18-β-GCA (0.1 mM), did not reduce the protective effect of αCT1 in ARPE-19 cells; neither did two modulators of HC-related activity, the ATP inhibitor apyrase and the microtubule binding domain Cx43 mimetic, JM2. These initial studies implicated a channel-independent role of the Cx43 CT in the regulation of barrier function, while the specific mechanism of action has yet to be determined.

A number of studies shed light on the potential channel-independent roles of Cx43 in the regulation of barrier function. These studies indicate that connexins influence cytoskeletal dynamics and cell-cell contact arrangements [19,20]. Cx43 has been shown to associate with the cytoskeleton, through direct interactions between its CT and the actin-binding proteins ZO1 and drebin [159,160], as well as microtubule proteins α- and β-tubulin [161,162]. In the context of wound healing scratch assays, brain endothelial cell monolayers treated with αCT1, resulted in a pronounced breakdown and misalignment in actin SFs, an effect requiring the peptide’s interaction with the PDZ2 domain of ZO1. The peptide-induced changes were accompanied by migratory effects, which were opposite those resulting from increased Cx43 expression [163]. Microtubule structure remained relatively unaffected in this study. Taken together, these experiments implicate the actin-cytoskeleton in potential channel-independent roles of the Cx43 CT and the Cx43 CT/ZO1 interaction in barrier modulation.

### 5.2. Connexins, Actin, and Potential Implications for Barrier Function Regulation

Several studies have shown associations between connexins, ZO1, and the dynamic actin cytoskeletal network associated with barrier function control. For example, Cx43 KO in neural crest cells resulted in shortened and misaligned SFs. This effect was accompanied by a shift in actin phenotype to a polygonal array around the cell periphery, concomitant with a decrease/disruption in vinculin and β1-integrin-associated focal adhesions [164]. In another study, Cx43 KO mouse embryonic fibroblasts, compared to wildtype cells, exhibited random polarity in the alignment between the Golgi and microtubule-organizing center (MTOC) with respect to the leading edge of the cell. These changes were accompanied by a failure of actin SFs to align correctly [16]. Consistent with the effect of Cx43 KO on actin structures, overexpression of Cx43 in C6 glioma cells resulted in a reorganization of actin into SFs [165]. Moreover, other studies have shown that expression of Cx43 or Cx32 resulted in upregulation of aligned actomyosin SF bundles [166,167]. These studies point to a critical role for Cx43 in the proper formation and alignment of the actin-based contractile machinery underlying barrier function control.

Just as the presence of Cx43 appears to support actin structure and function, an intact actin cytoskeleton seems to be a prerequisite to the localization and function of Cx43. For instance, inhibition of myosin II activity by blebbistatin in tenocytes reduced F-actin, while also reducing Cx43 [168]. This lead the authors to hypothesize that actin filaments might be required to stabilize GJ coupling during periods of intense mechanical loading and cellular stress. In line with this idea, another set of experiments demonstrated that viral-mediated expression of a 20 kDa Cx43 CT fragment caused an increase in F-actin polymerization, fiber length and thickness in cardiomyocytes [169,170]. In this study it was shown that microtubule-based trafficking of Cx43 to the membrane was stabilized in the presence of the 20 kDa isoform due to these effects on the actin cytoskeleton. Taken together, these experiments suggest a mutually dependent stabilization of cell border-located Cx43 and actin SF structure.

A role for ZO1 has been implicated in this relationship between Cx43 and actin. For instance, increased actin SFs induced by RhoA/ROCK signaling promoted association between ZO1 and Cx43 [129]. Another study showed that TAT-Cx43CT10, could overcome actomyosin contractility-induced inhibition of Cx43 HC activity [171]. TAT-Cx43CT10 is a cell-permeable peptide corresponding to the last 10 amino acids of the CT of Cx43 (SRPRPDDLEI), similar to αCT1 and TAT-CT9. This effect was lost with utilization of its reverse sequence (TAT-Cx43CT10Rev). αCT1 has been shown to target Cx43 function via direct Cx43 binding [29] in addition to its well-characterized property of inhibiting Cx43/ZO1 interaction as previously discussed. Given these studies, combined with the findings of Chen and co-workers [163], both Cx43 and an intact Cx43 CT/ZO1 interaction appear to correlate with proper formation, alignment, and function of actin structures involved in barrier function disruption.

On the other hand, Kameritsch and colleagues [172] conducted a study in HeLa cells, potentially implicating the Cx43 CT in barrier function protection. The authors showed that the full-length Cx43 CT (Cx43 aas 252–382), introduced into cells under normal conditions, enhanced filopodia formation (a cortical actin phenotype) via interaction and activation of PAK1. This interaction resulted in downstream p38 MAPK-mediated activation of actin capping protein Hsp27. Given that cortical actin phenotypes typically enhance barrier properties [107,111,173], this study raised the possibility that the Cx43 CT might facilitate an actin-regulatory signaling pathway that is conducive to barrier-stabilization. It turns out that the PAK1 signaling pathway overlaps with the actin SF-associated Rho/ROCK pathway. While both pathways involve actin polymerization, they diverge, in that the cortical actin pathway is downstream of Rac (often activated by PI3K, upstream of PAK1), which leads to activation of Abl-family kinases. Abl-family kinases stimulate actin polymerization through signaling via barrier-stabilizing cortactin [174]. Interestingly, a recent study reported that c-Abl is required for α5β1-integrin activation [175]. In this study, the c-Abl inhibitor nilotinib blocked α5β1-integrin. Activated α5β1-integrin has been shown to be required for connexin-based HC opening [176]. Thus, Cx43 appears to act as a signaling hub, supporting two processes involved in actin polymerization; one that could facilitate barrier function maintenance associated with HC closure, under normal circumstances; and another associated with barrier function disruption, under pathological conditions.

Alternatively, the cytoskeletal phenotype exhibited by Cx43 CT-expressing HeLa cells in the study by Kameritsch et al. [172], might be classified as barrier-disruptive, since the filopodial extensions appeared within areas of cell-cell contact at what could be considered to be an immature stage of barrier formation. Yet, since TJ-deficient HeLa cells cannot be classified as barrier-forming cells [177] and barrier function was not measured in this study, this would be speculation. What seems more concrete, however, is that actin fiber termini contained within filopodia-like cytoplasmic extensions represent an area typically occupied by a high density of focal adhesions [178], which serve as anchorage points for barrier-disruptive contractile actin fibers.

### 5.3. Cx43, Focal Adhesions, PI3K/PKCα Signaling, the Actin Cytoskeleton, and Barrier Function

A survey of the literature on Cx43′s connection to focal adhesions sheds light on the potential role of Cx43 in the modulation of actin-mediated barrier function. Cx43 has been shown to colocalize with vinculin and F-actin at points of actin fiber termination in cardiac neural crest cells [164]. These are sites of immature cell-cell contacts. A later study performed by Zemljic-Harpf and colleagues [71] demonstrated that Cx43, vinculin, and ZO1 not only colocalize at intercalated discs between cardiomyocytes, but that the three proteins co-immunoprecipitate with each other, while only vinculin and ZO1 interact directly. These studies raise the question of Cx43′s role at sites of integration between actin and focal adhesions in the regulation of barrier function.

Focal adhesion dynamics (initiation and disassembly) during pathological stress involves protein kinase α (PKCα) recruitment to focal adhesion complexes (e.g., by ZO1 PDZ2 binding partner PIP2) [179,180]. PKCα has been shown to associate with Cx43 and has been implicated as a major TPA-activated Cx43 kinase in R6-PKC3 fibroblasts [181], although the functional effect of this kinase on Cx43-based channel properties has not been conclusively determined. PKCα is a conventional PKC, which entails that its activation requires the presence Ca^2+^, among other molecules (e.g., DAG and phospholipids). PKCα has been shown to regulate vinculin/α-catenin binding in intestinal epithelial cells [179]. Multiple studies indicate that PKCα interacts with, phosphorylates, and activates vinculin while localizing to terminal SF endpoints in multiple cell lines, including embryonic fibroblasts [180,182]. Taken together, it appears likely that there are pathological stress conditions under which PKCα, Cx43, ZO1, vinculin, and actin colocalize with focal adhesions to regulate barrier-associated actin dynamics.

PKCα activation by various inflammatory stimuli (e.g., thrombin and TPA) has been shown to disrupt TJs and mediate increases in hyperpermeability [183,184,185]. PKCα is a well-known downstream target of PI3K activation [186,187]. PI3K is a regulator of the RhoGTPase family and regarded as a central to modulation of barrier function dynamics [112,187,188,189]. One study reported a downregulation of TJ proteins ZO1 and occludin in response to PI3K activation in Caco-2 cells [190]. PI3K is known to activate the Rho/ROCK pathway, which triggers actin SF formation and contraction associated with the breakdown of cell-cell contacts [191]. Inhibition of PI3K with Ly294002 prevented TNF-α induced formation of focal adhesions and SFs [192].

While it has not been determined if and how Cx43 directly interacts with PI3K, the presence of Cx43 has been shown to associate with PI3K signaling in several studies [193,194,195]. Ishikawa and colleagues [196] showed that Cx43 downregulation reduced PI3K activation. In this study, Cx43 co-immunoprecipitated with the β-subunit of G proteins (Gβ) which activated PI3K, and knockdown of Gβ mimicked the effect of Cx43 knockdown on signaling mediators downstream of PI3K (e.g., AKT and GSK). Furthermore, αCT1 has been shown to downregulate the PI3K/AKT pathway in tumor cells [197]. Since αCT1 does not downregulate Cx43 but decreases the ratio of HC/GJ [38] while increasing S368 phosphorylation, downregulation of the PI3K/PKCα pathway might be causally a result of either: (1) downregulation of either HC or GJ communication; or a (2) disruption of an intact Cx43/ZO1 interaction. As mentioned in a previous study by Xu and colleagues [164], Cx43 knockout resulted in a downregulation of vinculin-based focal adhesions, concomitant with a loss of F-actin SF formation and alignment. Thus, barrier function modulation, accompanied by proper F-actin polymerization and alignment, might depend upon a PI3K/PKCα-mediated Cx43-based stabilization of focal adhesions.

Potential channel-independent and -dependent roles for Cx43 in the dynamics of focal adhesions and barrier function-associated actin structures extend further. Focal adhesions are mechanosensory complexes that respond to stress/strain signals from the environment via integration with actin cytoskeletal structures [198]. Cx43-based channel activity has been implicated in mechanosensing in multiple studies [199]. In osteocytes for instance, Cx43 HCs open en masse in response to fluid flow and were experimentally determined to mediate the effects of mechanical stimulation [200]. Not surprisingly, multiple studies have implicated PKCα in transduction of mechanical stimulation of osteoblast cells [201,202]. A study by Batra and colleagues [176] showed that PI3K signaling resulted in shear stress-induced conformational activation of α5β1 integrin. Moreover, PI3K activation in this study triggered an interaction between α5β1-integrin and Cx43, resulting in the opening of Cx43 HCs. A recent study reporting in silico (computational and mathematical), ex vivo, and in vitro evidence of scar formation demonstrated that αCT1 may render cells insensitive to biomechanical inputs during scar differentiation [203]. Computational modeling predicted that αCT1′s alteration of collagen organization, resulting from its effects on fibroblast migration characteristics (as fibroblast secrete collagen), occurs in a mechanically-sensitive manner. αCT1-treated fibroblasts exhibited random directionality of migration as well as alteration in tail retraction during cell migration. Actin SF formation and contraction are required for tail retraction during directional migration [204,205]. Thus, all evidence considered, it appears that the HC-dependent activity of Cx43 and/or an intact Cx43/ZO1 interaction at focal adhesions might support actin SF formation and alignment. The intimate link between Cx43 and actin could ultimately regulate the actomyosin pulling forces exerted through ZO1 as tension transducer, onto AJ and TJ-based cell-cell contacts in a PI3K/PKCα-dependent manner. An integrative model for potential channel-dependent and -independent roles of Cx43 in actin-mediated barrier function pathology is proposed in Figure 3 (Figure 3A–C). Future experiments might directly investigate a potential role of Cx43 in a PI3K-mediated, cytoskeletal-associated modulation of barrier function. 

### 5.4. Cx43, Cell Chirality, and Actin-Mediated Barrier Function Regulation

Evidence for Cx43′s involvement in actin-associated barrier function raises the prospect that it may also have implications for a newly characterized morpho-biological property, cellular chirality. Cell chirality drives the asymmetrical differentiation and development of cells, tissues, and organs [206,207]. Cell chirality is handedness (left or right or clockwise (CW) or counterclockwise (CCW)) across three directions. An object is considered chiral when its mirror images cannot be superimposed (e.g., left and right hand). Since most biological molecules are chiral, it is thought that they serve as the foundation from which the chirality of cells, organs, and even larger biological structures (e.g., hands) are derived. Endothelial and epithelial cells often achieve their chirality by exhibiting polarity at all three of their axes: left-right axis, apical-basolateral axis, and front-rear axis. Wan and colleagues [208,209] established that cell chirality is intrinsic to cells and is very much a universal property shared across mammalian species (epithelial and endothelial cells). Cell chirality is largely determined by the alignment and structural modifications of the actin cytoskeleton [210]. A number of studies have indicated that the physical origins of chirality lie in the actin cytoskeletal dynamics, involving left-right symmetry breaking by actomyosin torque generation [211], the unidirectional arrangement of α-actinin [210], the right-handed spiral motor of myosin and the right handedness of the actin helix [212]. 

Interestingly, a recent pioneering study by Fan and colleagues [213] showed both a correlation and a causal relationship between cell chirality and barrier function. They found that endothelial barrier disruption triggered by an intermediate dose of the PKC activator IndoV, correlated to reduced ZO1 expression at the cell border and a reversal of cell chirality(from the CW direction to CCW direction) in a PI3K/PKCα-dependent manner. In this study, they demonstrated that the degree to which neighboring cells were misaligned with each other, directly correlated with the magnitude of barrier disruption and ZO1 downregulation. These changes were blocked with inhibition of both PKCα and PI3K. Using a panel of cytoskeletal inhibitors (e.g., Lat-A, Cytochalasin D, Jasplakinolide, Nocodazole), it was shown that the alteration of actin, but not microtubule function, was involved in these chirality changes. 

Cell-cell contact alignment has been shown to be affected by connexin’s association with the cytoskeleton [19]. GJ structures link to actin and microtubule organizing centers via direct interactions with ZO1 and α- and β-tubulin, respectively. Several studies have shown Cx43′s involvement in cell polarity [164,214,215,216]. A role of connexins in cellular chirality dates back to the 1990′s in an attempt to explain the unidirectionality of the propagation of electrical conduction in the heart [217]. It was found that in human patients with isomerisms, or deficits in LR asymmetry in the heart, there were mutations in several of the Cx43 serine motifs involved in the regulation of a Cx43/ZO1 interaction and GJ assembly (e.g., S364, S365, and S373) [218,219]. It must be noted, however, that the role of Cx43 in lateralization deficits in human patients has been disputed [220]. Nevertheless, all evidence considered, these studies indicate the possibility that Cx43 and/or a Cx43/ZO1 interaction participate in chirality-associated changes in barrier function (Figure 3C). Future experiments might be conducted to further probe the role of Cx43 in cell chirality-associated barrier function dynamics.

## 6. Conclusions

Evidence for channel-dependent roles of Cx43-based GJs and HCs in barrier function have mounted over the last 20 years as connexin-based channels can transmit barrier-disruptive signals. Channel-gated Ca^2+^ transfer and ATP release are involved in downstream cytoskeletal changes associated with barrier function modulation. PI3K/Rho/ROCK/MLC actin-modulatory pathways intersect with PKCα signaling to implicate a role of Cx43 and the Cx43/ZO1 interaction in the modulation of focal adhesions and the actin cytoskeleton. This potential mechanistic link between Cx43 and the cytoskeleton would likely result in alterations of AJ-based cell-cell contacts and TJ-regulated barrier function. Multiple studies have employed Cx43 mimetics (Gap19, Gap 26, Gap27, JM2 and αCT1) as tools, revealing homeostatic mechanisms underlying a role for connexins in barrier function. These studies, combined with an examination of the literature linking Cx43 to the actin cytoskeleton, suggest that channel-dependent and -independent roles of Cx43 could overlap with respect to an actin cytoskeletal-mediated regulation of barrier function.
